# International consensus on the use of tau PET imaging agent ^18^F-flortaucipir in Alzheimer’s disease

**DOI:** 10.1007/s00259-021-05673-w

**Published:** 2022-01-03

**Authors:** Mei Tian, A. Cahid Civelek, Ignasi Carrio, Yasuyoshi Watanabe, Keon Wook Kang, Koji Murakami, Valentina Garibotto, John O. Prior, Henryk Barthel, Rui Zhou, Haifeng Hou, Xiaofeng Dou, Chentao Jin, Chuantao Zuo, Hong Zhang, Molecular Imaging-based Precision Medicine Task Group of A3 (China-Japan-Korea) Foresight Program

**Affiliations:** 1 PET Center, Huashan Hospital, Fudan University, Shanghai, 200235 China; 2 Department of Nuclear Medicine and PET Center, The Second Affiliated Hospital of Zhejiang University School of Medicine, 88 Jiefang Road, Hangzhou, 310009 Zhejiang China; 3 Human Phenome Institute, Fudan University, Shanghai, 201203 China; 4 Department of Radiology and Radiological Science, Johns Hopkins Medicine, Baltimore, MD 21287 USA; 5 Department of Nuclear Medicine, Hospital Sant Pau, Autonomous University of Barcelona, 08025 Barcelona, Spain; 6 Laboratory for Pathophysiological and Health Science, RIKEN Center for Biosystems Dynamics Research, Kobe, Hyogo 650-0047 Japan; 7 Department of Nuclear Medicine, Seoul National University College of Medicine, Seoul, 03080 South Korea; 8 Department of Radiology, Juntendo University Hospital, Tokyo, 113-8431 Japan; 9 Diagnostic Department, University Hospitals of Geneva and NIMTlab, University of Geneva, Geneva, Switzerland; 10 Department of Nuclear Medicine and Molecular Imaging, Lausanne University Hospital, Lausanne, Switzerland; 11 Department of Nuclear Medicine, Leipzig University Medical Center, Leipzig, Germany; 12 National Center for Neurological Disorders & National Clinical Research Center for Aging and Medicine, Huashan Hospital, Fudan University, Shanghai, 200040 China; 13 Key Laboratory of Medical Molecular Imaging of Zhejiang Province, Hangzhou, 310009 China; 14 The College of Biomedical Engineering and Instrument Science of Zhejiang University, Hangzhou, 310007 China; 15 Key Laboratory for Biomedical Engineering of Ministry of Education, Zhejiang University, Hangzhou, 310007 China

**Keywords:** ^18^F-flortaucipir, Alzheimer’s disease (AD), Positron emission tomography (PET), Brain imaging

## Abstract

**Purpose:**

Positron emission tomography (PET) with the first and only tau targeting radiotracer of ^18^F-flortaucipir approved by FDA has been increasingly used in depicting tau pathology deposition and distribution in patients with cognitive impairment. The goal of this international consensus is to help nuclear medicine practitioners procedurally perform ^18^F-flortaucipir PET imaging.

**Method:**

A multidisciplinary task group formed by experts from various countries discussed and approved the consensus for ^18^F-flortaucipir PET imaging in Alzheimer’s disease (AD), focusing on clinical scenarios, patient preparation, and administered activities, as well as image acquisition, processing, interpretation, and reporting.

**Conclusion:**

This international consensus and practice guideline will help to promote the standardized use of ^18^F-flortaucipir PET in patients with AD. It will become an international standard for this purpose in clinical practice.

## Introduction

The prevalence of dementia worldwide has been estimated at 24 million and is expected to double every 20 years or more until 2040 [1]. Alzheimer’s disease (AD) is the most common cause of dementia, accounting for 60–80% of dementia cases [2–4], and affects approximately 6% of the population over the age of 65 [5]. According to the National Institute on Aging and Alzheimer’s Association (NIA-AA), AD has been defined for research purposes by its underlying pathological processes that may be documented by postmortem or in vivo biomarker examination, shifting the definition of AD in living people from a syndromal to a biological construct [6]. The NIA-AA adopted the proposition of “A/T/N system” in which three categories of AD biomarker (imaging and biofluids) are summarized based on the nature of the pathologies mechanisms: A (biomarkers of β-amyloid, Aβ)/T (biomarkers of fibrillar tau)/N (biomarkers of neurodegeneration or neuronal injury) [7]. The NIA-AA agreed that only biomarkers that are specific to characteristic AD proteinopathy (i.e., Aβ and tau) should be considered potential biomarker definitions of the disease.

The biomarkers of Aβ are the first found to become abnormal in AD [8]. According to the Aβ cascade hypothesis, abnormal production and accumulation of Aβ in the brain are the primary influences driving AD pathogenesis [9]. The in vivo measurement of cerebral amyloidosis as obtained using ^11^C-PiB [10] or one of the approved ^18^F-labelled amyloid tracers and positron emission tomography (PET) is associated with cross-sectional regionally specific brain atrophy and longitudinal cognitive decline. PET imaging of amyloid as target has also been extensively used in clinical trials testing anti-amyloid drugs [11]. The research advances in AD pathogenesis and the repeated failures of clinical trials for the anti-amyloid drugs further motivated a focus shift to investigate the role of tau pathology in the AD pathogenesis, also as target for novel drug developments [12]. Human and animal model data suggest a causal upstream role for Aβ in the pathogenesis of AD, but Aβ alone is insufficient to cause cognitive deterioration directly [13]. Postmortem studies also found that tau, but not Aβ, correlates to the severity of dementia and neurodegeneration, suggesting a more direct impact of tau aggregation on neurodegeneration than Aβ [14]. However, the role of tau in the pathophysiology of AD and other tauopathies remains unclear. Consequently, the precise targeting of tau deposition in vivo in the brain would be very valuable.

The key tools of “transpathology,” for example, the use of PET, can noninvasively detect the molecular markers in vivo and allow for an earlier disease diagnosis and evaluation than structural imaging methods [15]. In recent years, with the development of PET technology and the advent of new tracers, the pathophysiological changes of many major neurodegenerative diseases, including AD, have been explored with the help of PET molecular imaging [16]. There are several tracers for PET that have been developed and used for clinical assessment in patients with various tauopathies as “first-generation” tracers, such as ^18^F-THK5317 [17], ^18^F-THK5351 [18], ^18^F-AV-1451 [19], and ^11^C-PBB3 [20]. Limitations of these tracers with regard to off-target binding and diagnostic range provided the motivation to develop a new generation of tau tracers, including ^18^F-MK-6240, ^18^F-PI-2620, ^18^F-RO-948, ^18^F-JNJ311/069, ^18^F-GTP1, and ^18^F-PM-PBB3 [21–24].

In principle, tau PET imaging enables noninvasive detection of in vivo tau deposition patterns, facilitates differential diagnosis between neurodegenerative diseases including different tauopathies, and predicts disease progression. Furthermore, tau PET imaging could potentially be applied to achieve the therapeutic effect evaluation of anti-tau treatment and develop a novel drug, thereby allowing preventive interventions. In May 2020, US Food and Drug Administration (FDA) approved the first tau PET tracer ^18^F-flortaucipir, representing a great achievement to improve AD diagnosis.

The current consensus summarizes the use of ^18^F-flortaucipir PET in the clinical setting, including the clinical indications, qualifications and responsibilities of personnel, imaging procedure/specifications, documentation/reporting of the result, imaging equipment specifications, quality control and improvement, imaging safety and infection control, and the patient education concerns and the radiation safety in imaging.

## Goals

**Fig. 1:**
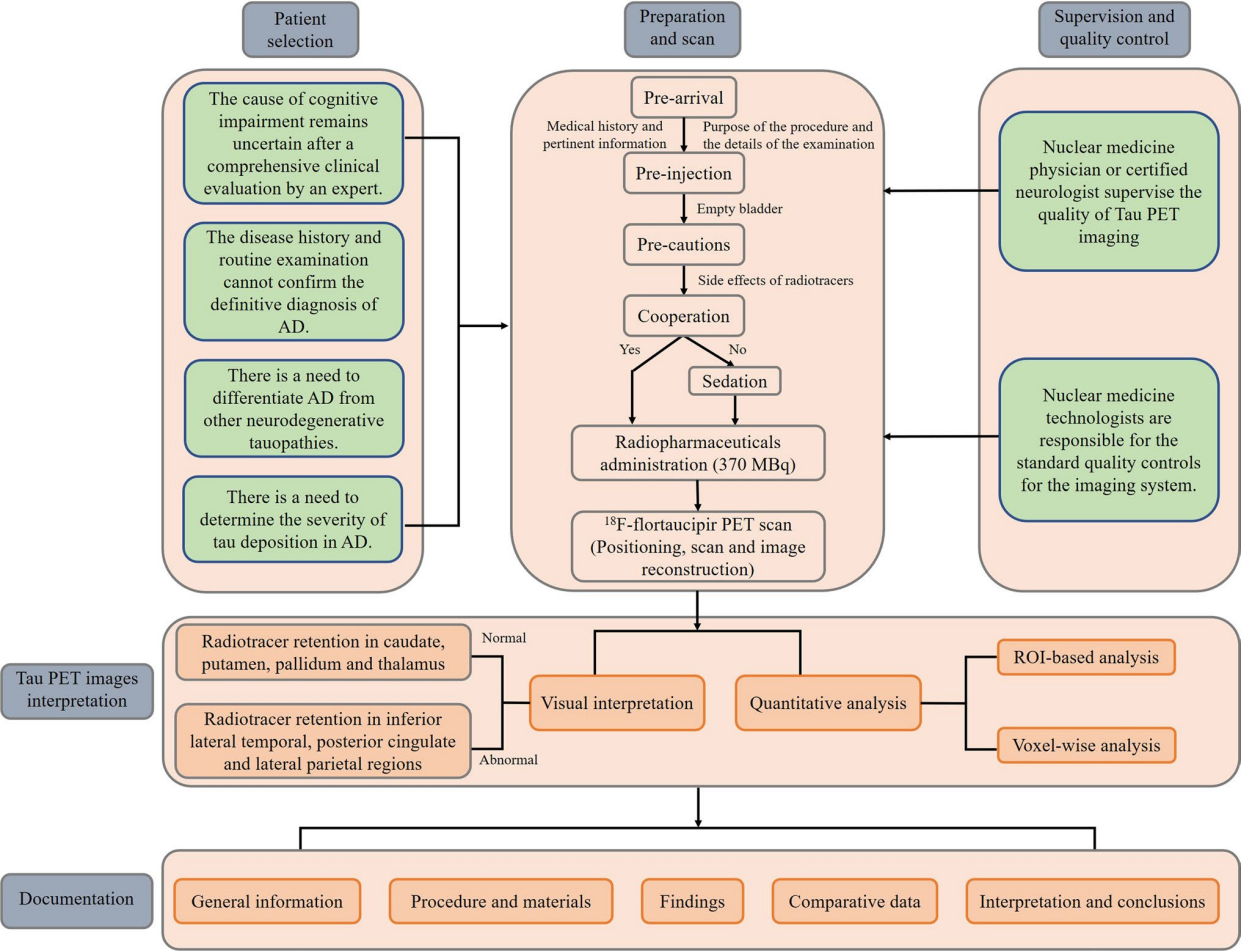
Flowchart for the recommended procedures in conducting ^18^F-flortaucipir PET scanning and reporting

The goal of this consensus is to provide a reference for nuclear medicine practitioners in procedurally preparing and performing ^18^F-flortaucipir PET imaging and properly interpreting and reporting the results of ^18^F-flortaucipir PET images (Fig. 1).

## Definitions

### Tau

Tau protein is a multifunctional protein mainly locating in axons of central nervous system, playing a crucial role in stabilizing microtubules [25]. There are six tau isoforms existing in human brain, classified as either 3 (3R) or 4 (4R) repeated binding domains, generated by alternative mRNA splicing of the microtubule-associated protein tau gene (MAPT) [26]. Several neurodegenerative diseases are characterized by tau aggregates; however, the tau aggregates vary in those disease either for morphology (e.g., neurofibrillary tangles for AD vs. astrocytic plaques, Pick bodies, or globose tangles in other neurodegenerative tauopathies) or ultrastructural conformations (e.g., paired helical filaments in AD vs. mainly straight or twisted filaments in other neurodegenerative tauopathies) [27].

#### AD

AD is an age-dependent neurodegenerative disease that is characterized as a slow start and progressive decline in memory, problem-solving, language, and other cognitive abilities, which is the main cause of dementia. The causes of AD are still unclear, while Aβ deposition and abundant tau phosphorylation are the main hallmarks of AD pathology.

### Mild cognitive impairment (MCI)

MCI is defined as a potential intermediate state between normal aging and dementia, with a reduction of cognitive performances but with preserved autonomy [28]. MCI was first proposed as stage III (mild cognitive aging) of global deterioration scale (GDS) for primary degenerative dementia in 1982 by Barry et al. [29]. Individuals at GDS stage III have slight cognitive deficits and may have some impairment in complex executive function. About 3–19% of individuals will develop MCI in natural aging, and the risk of progressing to dementia is 11–33% in 2 years [30].

### Neurodegenerative tauopathies

Neurodegenerative tauopathies are a group of dementia and movement disorders that are characterized as abundant accumulations of filaments assembled by microtubule-associated protein tau (MAPT), which includes AD, frontotemporal lobar degeneration (FTLD), corticobasal syndrome (CBS), and progressive supranuclear palsy (PSP). Though the symptoms of these diseases are different, brain abnormalities and cognitive impairment are associated with the growing accumulation of filaments or tangles.

### Tau imaging

Tau PET imaging could allow obtaining tau pathology information non-invasively in vivo. As such, it should be capable of providing the “T” biomarker information to support establishing a biological diagnosis of AD and other tauopathies, potentially already at earlier disease stages, like in MCI. Notably, ^18^F-flortaucipir presents higher affinity to “AD-like” tau aggregates than that in other neurodegenerative tauopathies, while it should be noticed that the sensitivity of ^18^F-flortaucipir might not be enough to diagnose early stage of AD, such as Braak stage 0.

## Clinical indications

To our knowledge, there is no consensus or recommendation on indications/contraindications for the clinical application of tau PET imaging. Despite tau PET remains as a powerful research tool in tauopathies, we suggest to consider the use of ^18^F-flortaucipir PET imaging in the following clinical scenarios: the cause of cognitive impairment remains uncertain after a comprehensive clinical evaluation by an expert; the disease history and routine examination cannot confirm the definitive diagnosis of AD (as it would be possible, e.g., in familial AD); there is a need to differentiate AD from other neurodegenerative tauopathies; there is a need to determine the severity of tau deposition in AD.

The ^18^F-flortaucipir PET is not recommended to be clinically used to evaluate tauopathies other than AD (e.g., in other neurodegenerative tauopathies) [31], and the sensitivity of ^18^F-flortaucipir PET is limited in detecting early-stage tau pathology [32]. Additionally, it should be careful to conduct longitudinal assessment by using ^18^F-flortaucipir PET, as more evidence is required to support the value of ^18^F-flortaucipir PET imaging in longitudinal assessment.

## Qualifications and responsibilities of personnel

### Physician

Neurology experts should prescribe the tau PET examination only when appropriately indicated. An adequately trained nuclear medicine physician is responsible for the report and supervision of the quality of tau PET imaging. With the authorization of local, state, and national regulations, this procedure could also be performed and supervised by a certified neurologist with required additional radiation safety and nuclear medicine training.

### Technologist

Tau PET imaging procedure should be performed in a certified imaging facility and operated by a qualified nuclear medicine technologist who has appropriate training and certification. The operation should be performed under the supervision of a physician as mentioned, in compliance with existing standard operating policies and procedures at the imaging facility. Please also see references [33, 34] for general requirements of nuclear medicine technologists.

### Medical physicist/technologist

A qualified medical physicist expert should ensure the safe use of radiotracers, monitor radiation dose, and develop image acquisition and reconstruction algorithms, as well as to better protect the patient and staff alike. Further details can be found in the American College of Radiology, ACR-AAPM technical standard for medical physics performance monitoring of PET/CT imaging equipment, and the European guidelines on medical physics expert could provide more details [35, 36].

## Procedure/specifications of the examination

### Patient preparation

#### Pre-arrival

Before the start of the procedure, medical history and pertinent information on the patient should be obtained. It is recommended to carefully explain the purpose of the examination and the details of the procedure to the patients, which would reduce their anxiety and results in a better cooperation during the procedure. Patients do not need to fast as food intake does not interfere with ^18^F-flortaucipir PET imaging. There is no evidence that antidementia drug has an effect on ^18^F-flortaucipir. Therefore, it is not recommended to withdraw the drug before ^18^F-flortaucipir PET imaging. When applicable, patients are recommended to arrive at the hospital with an accompanying person.

#### Pre-injection

All the possible risks should be discussed with the patients, and signature of an informed consent should be performed when applicable. Patients with severe cognitive impairment should be accompanied by a caregiver. Patients need to empty their urinary bladder before scanning to maximally reduce the radiation.

#### Precautions

General precautions should be conducted to avoid injection-related infection, radiotracer infiltration, and radiotracer spillage. The baseline activity of the ^18^F-flortaucipir should be determined by using an appropriately calibrated dose calibrator before the administration of the radioactive ‘dose’.

Specific precautions should be considered for ^18^F-flortaucipir PET imaging. It is reported that the administration of ^18^F-flortaucipir might occasionally lead to adverse events, such as headache, injection site pain, and gastrointestinal disorders [37]. Therefore, the patients and family members or caregivers should be instructed to inform the physician when such symptoms occurred.

#### Sedation

Sedation may be exceptionally required for patients who cannot cooperate with the examination. The method might be adjusted for individual patients and modified by the physician. Sedation should be conducted just before the ^18^F-flortaucipir PET examination so that all the procedures will proceed without delay.

### Radiopharmaceuticals and administered activities

Among the tau targeting radiotracers investigated, ^18^F-flortaucipir is the first and only PET tracer approved by FDA for clinical practice; we provide additional details of ^18^F-flortaucipir PET imaging here. The production processes of ^18^F-flortaucipir should comply with national and local regulations. The manufacturer performs the radiopharmaceutical quality control before its release to the imaging centers. Baseline activity of 370 MBq ^18^F-flortaucipir in a total volume of 1–2 mL is recommended to inject as a single intravenous slow bolus.

### Image acquisition protocol

#### Positioning

A supine position with suitable head support is preferred to reduce the potential head movement. Extreme neck extension or flexion should be avoided. The entire brain, especially the entire cerebellum, should be included in the view.

#### Static emission scan

The 20-min brain PET scan is performed after 80–100 min of ^18^F-flortaucipir injection. As this tracer does not ready in apparent steady state, the adherence of this image acquisition time window is of critical importance for inter- and intra-individual comparison of the PET imaging results. The brain PET images (4 frames: 4 × 5 min) are acquired in three-dimensional (3D) mode with appropriate data corrections after the tracer injection. The acquisition protocol may vary in different institutions based on their imaging protocols and on types of equipment and radiotracer used. Dynamic acquisitions with subsequent reconstructions with different time frames can be considered when appropriate.

#### Image reconstruction

PET images are reconstructed at least in a 256 × 256 image matrix size with attenuation correction. The typical pixel size is 2–4 mm with a slice thickness of 2–4 mm. The acquired images are recommended to be reconstructed by using 3D ordered subset expectation maximization (OSEM).

### Interpretation of the tau brain PET images

Image quality needs to be evaluated before interpreting the study to ensure the quality of ^18^F-flortaucipir PET images, such as the presence of the patient’s head motion. Any noted limitations that might compromise the image interpretation should be indicated in the report.

#### Visual interpretation of ^18^F-flortaucipir PET brain images

PET images are displayed in axial, coronal, and sagittal views and 3D-MIP images. Color scales are often used and might be selected according to availability and the reader’s preference. It is recommended to adjust the color bar until the display of bilateral striatum with appropriate background. The intensity scales, contrast, and background subtraction should be adjusted in order to obtain a predefined ratio with the cerebellar cortex used as reference [38]. The coregistration of ^18^F-flortaucipir PET with anatomical images such as computer tomography (CT) or magnetic resonance imaging (MRI) is recommended for the definition and the localization of ^18^F-flortaucipir in comparison with visual assessment of PET images only.

**Fig. 2:**
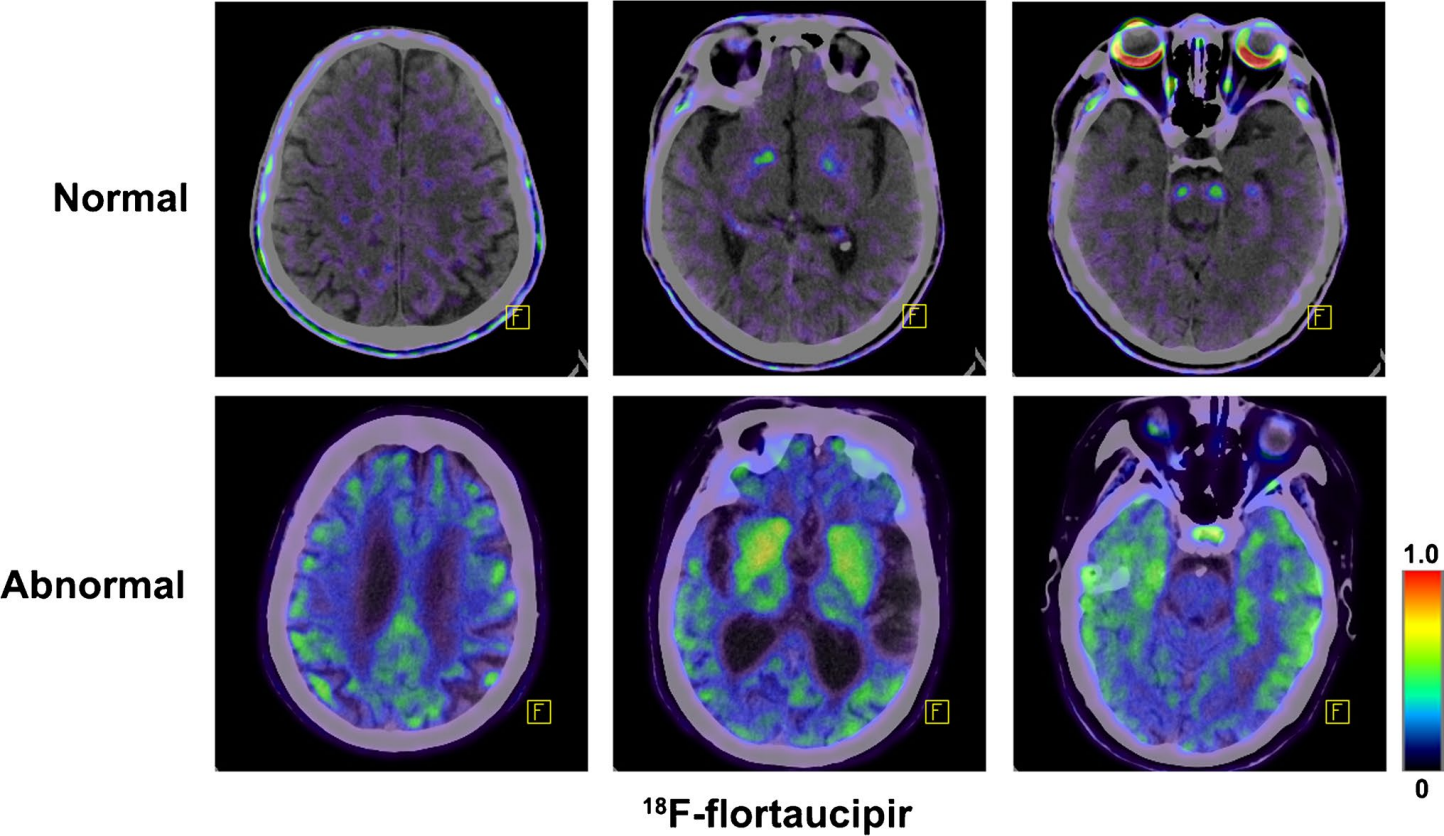
Representative ^18^F-flortaucipir PET images from a healthy elderly individual and patient with AD. The patient with AD showed marked radiotracer retention in bilateral temporal, temporoparietal, and posterior cingulate cortical regions

It is recommend to evaluate tau PET images from the bottom to the top in the perspective of transaxial plane. Cerebellar cortex could be used as negative control, since cerebellum is generally free of tau protein deposition. It is worth noting that ^18^F-flortaucipir retention could be observed in superior portion of the cerebellar gray matter [39]. Therefore, we recommend to use inferior cerebellar gray matter as negative control. Tau brain imaging using ^18^F-flortaucipir in cognitively normal (CN) young individuals shows few focal radiotracer retention. In elderly individuals with normal cognition, it shows no focal cortical retention but an age-related increased radiotracer retention in midbrain, caudate, putamen, pallidum, and thalamus as well as off-target binding in brainstem, striatum, and retina. Patients with AD have significant tau radiotracer retention in the advanced Braak stage brain regions, like the inferior lateral temporal, posterior cingulate, and lateral parietal regions (Fig. 2).

#### Quantitative analysis

##### SUVRs (standardized uptake value ratios)

SUVRs are widely used in clinical practice. Since the cerebellum region has less specific binding to tau protein, it can be used as the reference region. Volumes of interest (VOIs) of the Braak stage regions in case of suspected AD or of other brain regions depending on the suspected tauopathy allow to obtain target region standardized uptake values (SUVs), which will result in SUV ratios (SUVRs) when compared with the reference region SUVs.

##### Voxel-wise analysis

The voxel-wise analysis allows comparison of the difference between one subject and groups, or two groups directly in the whole brain after spatial and count rate normalization. The voxel-based analysis could detect subtle tiny abnormalities without the inherent restriction of predefined ROIs.

## Documentation/reporting

### General information

The report should include the patient demographics, identifiers, and all pertinent basic study information such as the name, age, gender, examination date, clinical diagnosis, net intravenously injected radioactivity of the administered radiotracer, and the i.v. injection site. The pertinent information associated with cognitive/motor impairment-related clinical symptoms should be included, ideally including respective cognition/motor symptom scores. Results of structural brain imaging, CSF biomarker testing in case of suspected AD, and of other potential nuclear brain imaging exams should also be provided. The reason for the test, such as the presence of uncertain clinical diagnoses, and whether there is a need to differentiate with non-AD tau pathologies, or presence of AD comorbidities, should be briefly described. Pertinent past medical history that might alter the anatomy of the brain, such as brain surgery, head trauma, and stroke, should also be briefly described.

### Body of the report

#### Procedures and materials

The type and the net administrated dose and the site of injected radiotracer should be documented and double-checked by the technologist and nurse. Any unwanted events that occurred during radiotracer injection, such as infiltration should be recorded.

#### Findings

Abnormalities should be described including the areas in which pathological cerebral and cerebellar uptake is observed. Performed quantitative or semi-quantitative assessments should be stated.

#### Comparative data

Comparisons with previous ^18^F-flortaucipir PET examinations and interpretations should be incorporated into the current study report. The findings of pertinent related recent imaging such as the brain MR, CT, ^18^F-FDG PET, amyloid PET, and neuropsychiatric examination findings should also be considered when interpreting ^18^F-flortaucipir PET images.

#### Interpretation and conclusions

The impression should state clearly whether the scan demonstrates positive or negative tau deposition. If the scan is inconclusive, this needs to be stated along with possible reasons, such as low counting rate, head motion during imaging, unexpected focal lesion, cortical atrophy, or other difficulties.

Notably, off-target binding of ^18^F-flortaucipir in the choroid plexus, brainstem, basal ganglia, and meninges has been reported [40]. Although ^18^F-flortaucipir PET imaging depicts tau deposition in the brain, it is crucial to note that a scan positive for tau deposition should be interpreted in association with amyloid PET imaging and clinical information, at present, to diagnose AD. Negative results of ^18^F-flortaucipir PET imaging accompanied with positive amyloid PET imaging indicate patients who are possibly developed with AD. Positive results of ^18^F-flortaucipir PET imaging combined with positive amyloid PET imaging indicate the existence of AD in the patients. Also, a positive ^18^F-flortaucipir PET result does not exclude other coexistent neurodegenerative disorders. On the contrary, negative results of amyloid PET imaging indicate patients who are unlikely to have AD and negative ^18^F-flortaucipir PET results among MCI patients may indicate that they are unlikely to advance to AD dementia [41, 42].

## Equipment specifications

Tau PET examinations can be performed on different PET systems from various manufacturers. Adequate knowledge of the technique and equipment used would help improving image quality and reducing imaging artifacts. To ensure optimal imaging performance, all scanners should undergo standard periodic quality control tests according to the manufacturer’s specifications. More detailed recommendations can be found on the Society of Nuclear Medicine Procedure Guideline for FDG PET Brain Imaging (Version 1.0), and EANM procedure guidelines for PET brain imaging using ^18^F-FDG (version 2.0) [43, 44].

## Quality control and improvement

Standard quality controls (QC) for each system have to be maintained according to manufacturer recommendations and national regulations. Preferably a qualified medical physicist supervises the establishment of the QC program, and an on-site technologist should be identified to conduct the defined routine QC procedures. See also Society of Nuclear Medicine Procedure Guideline for FDG PET Brain Imaging (Version 1.0), and EANM procedure guidelines for PET brain imaging using ^18^F-FDG (version 2.0) for details of PET QC [43, 44], and the ACR–ASNR–SPR practice parameter for the performance of computed tomography (CT) of the extracranial head and neck for details of CT QC [45].

## Safety, infection control, and patient education concerns

Policies and procedures on safety, infection control, and patient education should be developed and implemented according to regional and national regulations. For tau PET imaging, special attention should be paid to the safety issues of patients with dementia or other neurodegenerative diseases, in whom falls may frequently occur. Obstacles in the imaging department should be minimized, and patients should be carefully aided when walking in and out of the room. If patients would be restrained on the bed or in a head holder during the examination, frequent explanation and reassurance would help reduce the stress and anxiety. The presence of an accompanying familiar person can be helpful during the uptake phase and the installation of the patient in the scanner room provided general radioprotection of this person can be insured. General recommendations can be found in SNMMI Guideline for General Imaging [46], ACR Position Statement on Quality Control and Improvement, Safety, Infection Control and Patient Education [47], and SNMMI Procedure Standard/EANM Practice Guideline for Amyloid PET Imaging of the Brain 1.0 [48].

## Radiation safety in imaging

Following the principle of “as low as reasonably achievable (ALARA),” nuclear medicine physicians, technologists, and medical physicists have a responsibility to minimize radiation exposure to patients, staff, family members, and caregivers, as well as society as a whole, without degradation in image quality. As estimated by dosimetry studies for ^18^F-flortaucipir in clinical trials, the radiation exposure from a tau PET scan (about 4–9 mSv) is within the range of commonly performed other imaging studies. See also SNMMI Guideline for General Imaging, “EANM procedure guidelines for PET brain imaging using [^18^F]FDG, version 2”, and SNMMI Procedure Standard/EANM Practice Guideline for Amyloid PET Imaging of the Brain 1.0 [44, 46, 48].
